# Evidence Supporting the Uptake and Genomic Incorporation of Environmental DNA in the “Ancient Asexual” Bdelloid Rotifer *Philodina roseola*

**DOI:** 10.3390/life6030038

**Published:** 2016-09-06

**Authors:** Olaf R. P. Bininda-Emonds, Claus Hinz, Wilko H. Ahlrichs

**Affiliations:** AG Systematics and Evolutionary Biology, IBU–Faculty V, Carl von Ossietzky Universität Oldenburg, Carl von Ossietzky Strasse 9–11, 26111 Oldenburg, Germany; dr.claus.hinz@gmail.com (G.H.); wilko.ahlrichs@uni-oldenburg.de (W.H.A.)

**Keywords:** anhydrobiosis, asexual reproduction, Bdelloidea, desiccation, DNA uptake, genetic exchange, horizontal gene transfer

## Abstract

Increasing evidence suggests that bdelloid rotifers regularly undergo horizontal gene transfer, apparently as a surrogate mechanism of genetic exchange in the absence of true sexual reproduction, in part because of their ability to withstand desiccation. We provide empirical support for this latter hypothesis using the bdelloid *Philodina roseola*, which we demonstrate to readily internalize environmental DNA in contrast to a representative monogonont rotifer (*Brachionus rubens*), which, like other monogononts, is facultative sexual and cannot withstand desiccation. In addition, environmental DNA that was more similar to the host DNA was retained more often and for a longer period of time. Indirect evidence (increased variance in the reproductive output of the untreated F1 generation) suggests that environmental DNA can be incorporated into the genome during desiccation and is thus heritable. Our observed fitness effects agree with sexual theory and also occurred when the animals were desiccated in groups (thereby acting as DNA donors), but not individually, indicating the mechanism could occur in nature. Thus, although DNA uptake and its genomic incorporation appears proximally related to anhydrobiosis in bdelloids, it might also facilitate accidental genetic exchange with closely related taxa, thereby maintaining higher levels of genetic diversity than is otherwise expected for this group of “ancient asexuals”.

## 1. Introduction

Rotifera is a phylum of approximately 2000 named species of microscopic aquatic invertebrates, notable in part for the diverse reproductive strategies present within the group. These strategies characterize the major lineages within rotifers, with the major clades Monogononta and Bdelloidea demonstrating facultative sexual and obligate asexual reproduction, respectively, whereas species of the clade Seisonida, for which the phylogenetic affinities remain unclear (but see [1]), are obligate sexuals [2].

In this regard, the asexual bdelloids represent a puzzle (or “scandal” in the eyes of John Maynard Smith [3]) that has long confounded evolutionary biologists. Although the prevalence of the more costly sexual over asexual reproduction is still not understood fully [4], it remains that strict asexual reproduction entails many disadvantages that ultimately result in the extinction of asexual species over the mid to long term, thereby continuously limiting their numbers in nature [3,5,6]. Yet, despite hundreds of years of investigation, there has been no direct evidence of sex, meiosis, males or hermaphrodites [7,8] among any of the more than 450 extant species of bdelloid rotifers [9] and, by extension, among the more than 35 to 60 million years (but probably longer) of evolutionary history of the crown group as a whole [10,11].

The ability of most bdelloid species to enter and withstand anhydrobiosis at any life stage [12] is traditionally considered an important feature underlying the success of the group in the face of their asexual lifestyle [13]. They can survive in this dehydrated state for several weeks [14] during which wind dispersal enables the founding of new populations. In so doing, individuals can potentially escape unfavorable circumstances in space and time [15], whereby their genomes and attendant genetic load might have driven them to extinction otherwise [16].

Importantly, a pair of recent studies [11,17] propose a functional link between desiccation tolerance in metazoans and higher-than-normal levels of horizontal gene transfer (HGT) in these same lineages, which could function as a surrogate for genetic exchange in the absence of sex. Essentially, both studies suggest the same general mechanism: during rehydration following a desiccation event, the porous nature of cell membranes potentially facilitates the entry of large macromolecules like foreign DNA into the cell [18,19,20], and this DNA can then accidentally become incorporated into the genome, which is shredded during the desiccation process and needs to be reassembled [21,22,23] (for a more detailed description, see [17]).

Eyres et al. [11] also provide indirect support for this hypothesis by documenting higher levels of HGT in bdelloid species that regularly undergo desiccation events, suggesting thereby that HGT remains an on-going process in the group and an important mechanism of genetic exchange (also [24]). Indeed, the relatively high levels of HGT documented within bdelloid rotifers (about 8% of the genes in the genome or transcriptome of two species of *Adineta* [25,26] and 9.5%–14.2% in the transcriptomes of four species of *Rotaria* [11]) might be an important source of genetic diversity in this group of ancient asexual organisms as is also hypothesized to be the case in bacteria [27], possibly together with allele sharing facilitated by an atypical form of meiosis [28].

Although the empirical basis behind the conjecture of Boothby et al. [17] has been refuted (the tardigrade *Hypsibius dujardini* does not show unusually high levels of HGT [29]), the same mechanism was suggested by Eyres et al. [11] and could still apply for bdelloid rotifers. In this context, we explicitly test this general mechanism by examining the ability of the bdelloid rotifer, *Philodina roseola*, to take up environmental DNA and to incorporate it into its genome during a desiccation event. Although this capacity has been hinted at previously in bdelloid rotifers [30,31], there has been no direct evidence of it apart from the HGT events inferred for this group [11,24,25,26,32], which were assumed to have occurred during or following desiccation when the gut membranes are disrupted [32]. However, if the mechanism does occur in bdelloid rotifers, the role of HGT as a source of genetic diversity would then represent a beneficial by-product of the desiccation tolerance of the group. Importantly, the mechanism as put forth by both Boothby et al. [17] and Eyres et al. [11] yields two testable predictions:

1. Bdelloid rotifers (represented here by *P. roseola*) should internalize environmental DNA more readily than the monogonont rotifers (represented by *Brachionus rubens*), which cannot withstand desiccation (except as sexually produced resting eggs).
2. Incorporation of environmental DNA into the genome of *P. roseola* can only occur in conjunction with a desiccation event and should result in heritable changes in the untreated F1 generation (e.g., changes in fitness).

If verified, this process could provide a potential route of genetic exchange in bdelloid rotifers underlying increasing, albeit indirect evidence of recent HGT events [11,24] and thereby a possible explanation as to how this clade of ancient asexuals might have survived for so long with a reproductive strategy that otherwise represents an evolutionary dead end.

## 2. Materials and Methods 

### 2.1. Rotifer and Algal Cultures

Our *P*. *roseola* colony descends from a continuously hydrated culture that started from a single egg in 1989. Thus, although clonal in origin, accumulation of mutations over these ~1500 generations means that some (minor) genetic variation should now be present. The colony was cultured under a 9/15 h light/dark regime in ten different 90 × 15 mm plastic Petri dishes at 20 ± 1 °C, with weekly transfers to new Petri dishes. The algae *Cryptomonas* sp. SAG 26.80 was used as the food source and was cultivated separately under continuous illumination (13W/840 Osram Daylight neon tubes; Osram; Garching, Germany) in 500-mL Erlenmeyer flasks with COMBO medium [33]. A second bdelloid species, *Adineta ricciae*, was cultured similarly as was the facultative asexual monogonont rotifer *Brachionus rubens*, apart from being fed the algae *Monoraphidium minutum* SAG 243-1; only two Petri dishes were used for each of the latter two rotifer species.

### 2.2. Quantifying DNA Uptake

The ability of *P*. *roseola* to take up radioactive, environmental DNA was tested in parallel in both hydrated individuals as well as those that had been desiccated for one week. For the latter, individuals were placed in 2-mL Eppendorf tubes in 1 µL of medium and with a 1-cm^2^ piece of KIMTECH Science© delicate task wipe (Kimberly-Clark GmbH; Koblenz, Germany). Desiccation then followed for 15.0 ± 1.5 h at 40% relative humidity for seven days in a humido-thermostatic chamber custom-built by the University of Oldenburg workshops (= Protocol D of Ricci et al. [34]). Our desiccation survival rates, whether for isolated individuals or groups, of 88.5% match those obtained by Ricci et al. (88.2%). Tubes with 75 individuals each were incubated with conspecific DNA for either 3 or 15 h (10 versus 30 tubes, respectively); for the desiccation treatments, environmental DNA was added during the reanimation process. Twenty batches of 80 individuals of *B*. *rubens*, each similarly exposed to DNA for either 3 or 15 h, were used as a control.

Thereafter, the differential ability of *P*. *roseola* to take up alien DNA was tested in hydrated individuals using environmental DNA obtained from *B*. *rubens* (for 1.5 or 15 h), *A*. *ricciae* (15 h), or the additional monogonont species *Euchlanis dilatata* (15 h; individuals obtained from the Ems-Jade Channel near Mariensiel, Northwest Germany; N 53.0991º, E 8.1003º). All tests (each comprising 15 tubes of 75 individuals each) were run in parallel, with the uptake of environmental DNA from each donor species being compared to that from conspecific (*P*. *roseola*) DNA as a reference point.

All source DNA was obtained from batches of 70 individuals, with algae and other contaminants filtered out using a 60-µm mesh. Afterwards, animals were transferred into sterile COMBO medium, washed two times (with as much medium as possible being replaced with fresh, sterile COMBO medium each time), and left for two hours before being washed again. DNA was extracted using a 300-µL digestion solution of 10% Chelex® (Bio-Rad; Munich, Germany) and 0.07 µg·µL^−1^ of proteinase K. Samples were incubated for 30 min at 55 °C, followed by a 10-min heat-inactivation step at 95 °C before being cooled for at least 30 min at 5 °C. Following sedimentation of the Chelex® at 15,000 rpm for 15 s, the clear supernatant was transferred into sterile Eppendorf tubes and stored at −18 °C until use. DNA was labelled in vitro using the standard assay from the High Prime DNA Labelling Kit (Roche; Mannheim, Germany) using [alpha-^32^P]dCTP (Hartmann Analytic; Braunschweig, Germany); unincorporated dNTPs were removed with Sephadex G-50 (fine) QuickSpin columns (Roche; Mannheim, Germany).

For all experiments, radioactive DNA with 25,000 ± 473 counts min^−1^ was added to 2-mL test tubes (Qiagen; Hilden, Germany) each containing 75 individuals of *P*. *roseola* or *B. rubens* in 200 µL of algae-free COMBO medium; this procedure also served to reanimate the desiccated individuals. At the end of the respective incubation times, the tubes were placed on ice for 10 min followed by the washing out of any non-internalized DNA. Following centrifugation for 6 min at 0 °C and 9,500 rpm using a Heraeus Biofuge Fresco (Thermo Fischer Scientific GmbH, Dreieich, Germany) to momentarily stop the animals from swimming, all but 20 µL of fluid in each test tube was removed and 400 µL of fresh medium (without DNA or algae) was added. Centrifugation and subsequent washing was repeated six times. After the last centrifugation step, 300 µL of digestion solution instead of medium was added and DNA was extracted as described above. Radioactivity was measured in a Wallac® 1415 Scintillation counter (Perkin Elmer; Hamburg, Germany) using 20-mL PE-vials (Perkin Elmer; Hamburg, Germany) containing 200 µL of the supernatant from the DNA extraction mixed with 5 mL of LumaSafe (Lumac LSC; Groningen, The Netherlands). For each test, two tubes containing only COMBO-medium and marked DNA from the different sources were used to measure and correct the results for background radioactivity that could not be removed by washing.

### 2.3. Quantifying Reproductive Output

**Figure 1:**
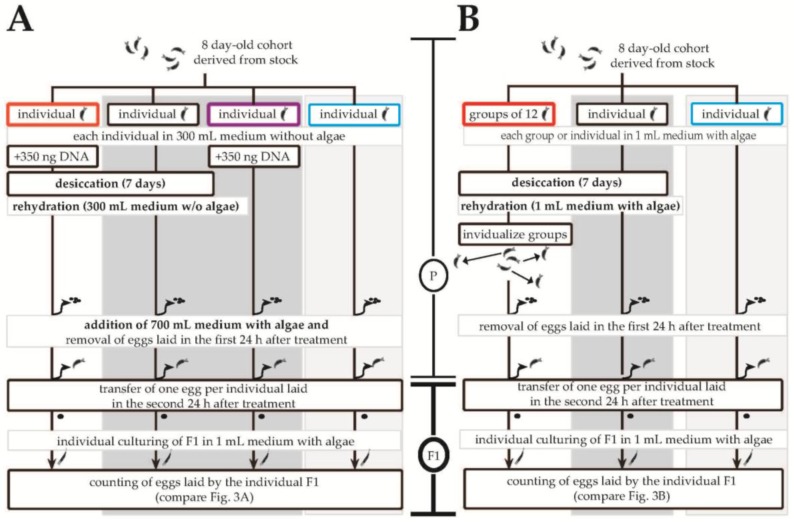
Experimental procedure to quantify changes in reproductive output of the untreated F1 generation of *Philodina roseola*. Factors examined were whether the parental generation was held in groups or individuals, whether or not it underwent a desiccation event, and whether or not conspecific, environmental DNA was added to the medium. According to the mechanism proposed by both Boothby et al. [17] and Eyres et al. [11], only populations in the first, unshaded columns of each of (**A**) and (**B**) should display heritable effects indicating the genomic incorporation of environmental DNA. The dark grey, shaded columns thus act as controls, whereas the light grey, shaded columns represent the reference populations.

Using the number of eggs laid as a proxy for fitness, we tested if internalized DNA influenced the variance of the reproductive output of the offspring as predicted by sexual theory (see [35,36]) and therefore had heritable effects by being incorporated into the genome. Individuals of *P*. *roseola* were desiccated either in groups with no additional DNA or individually with *P*. *roseola* DNA (unlabelled) added to the culture medium before desiccation. For both trials (Figure 1), offspring from continuously hydrated parents were used as a reference with additional controls for the effects of DNA addition without desiccation (hydrated individuals with DNA) and for desiccation itself (desiccated individuals without DNA). For each treatment, control and reference group, 96 eight-day-old adult individuals formed the parental generation. They were maintained in 24-well flat-bottom plates (Falcon® 353935; Corning; Wiesbden, Germany) with an algal density of around 10^6^ cells mL^−1^. Plates were desiccated for seven days in humido-thermostatic chambers custom-built by the electronic workshop of the University of Oldenburg. One egg from each parental individual contributed to the F1 generation, where the number of eggs laid was counted in 24-h intervals before being removed.

## 3. Results

### 3.1. Uptake of Environmental DNA

**Figure 2:**
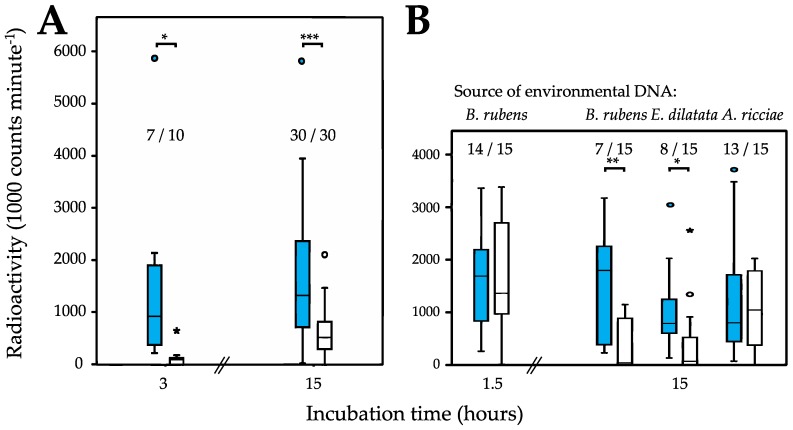
Pairwise comparisons of the uptake of radioactively labelled DNA. (**A**) Uptake of conspecific, environmental DNA by desiccated (white) compared to continuously hydrated (blue) individuals of *Philodina roseola*. (**B**) Uptake by desiccated *P. roseola* of environmental DNA obtained from the monogonont rotifers *Brachionus rubens* or *Epiphanes dilatata* or the bdelloid rotifer *Adineta riccae* (white) compared to conspecific DNA (blue). Significant pairwise differences in radioactivity levels are indicated (see also Table 1). Numbers above the bars indicate the proportion of pairwise trials of 75 individuals each in which any DNA was taken up in the white trials.

Continuously hydrated individuals of *P*. *roseola* display a remarkable ability to take up environmental DNA, one that was consistently shown in all trials even after comparatively short incubation times (e.g., 1.5 or 3 h; Figure 2 and Table 1). This finding is in stark contrast to that for the monogonont rotifer *Brachionus rubens*, which did not internalize any environmental DNA even after 15 h. The ability to take up DNA was also present, albeit reduced, in individuals of *P*. *roseola* that were desiccated for one week before being reanimated in the presence of environmental DNA (Figure 2A). Here, DNA was taken up in fewer trials (although not significantly fewer) compared with hydrated individuals and always at significantly reduced levels. Taken together, our results for these two species show that environmental DNA appears to be actively taken up by *P*. *roseola* and is not simply entering by chance sometime during the desiccation process.

DNA uptake appears to be unselective initially (Figure 2B), with hydrated individuals of *P*. *roseola* internalizing conspecific (*P*. *roseola*) and alien (*B*. *rubens*) DNA equally after 1.5 h, both in terms of frequency and amount. Thereafter, however, conspecific DNA is retained preferentially. After 15 h of incubation, both the number of trials in which *B*. *rubens* DNA was detected in *P*. *roseola* as well as the overall amount were significantly reduced compared with values for conspecific DNA after the same time or to the initial values for *B*. *rubens* DNA after 1.5 h. Similar results to those for *B*. *rubens* DNA were also found for donor DNA from a second monogonont species (*Euchlanis dilatata*) after 15 h. By contrast, uptake parameters compared with conspecific DNA were not significantly reduced for donor DNA from the bdelloid *Adineta ricciae*, suggesting strongly that donor DNA is somehow being filtered according to its overall similarity with that of the host animal, with more similar DNA being preferentially retained.

### 3.2. Genomic Incorporation and Fitness Effects

**Figure 3:**
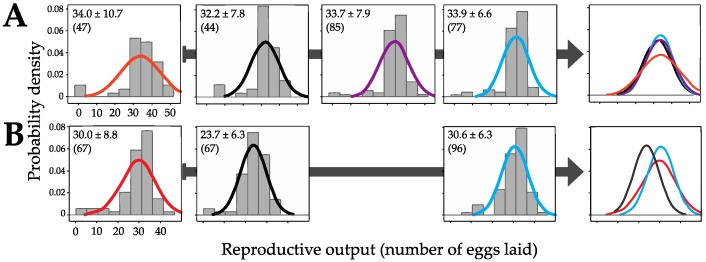
Changes from twin experiments in the reproductive output of the untreated F1 generation of *Philodina roseola* (see Figure 1). The trials in (**A**) and (**B**) correspond to those in Figure 1A,B, respectively, both in their ordering and colors. The rightmost set of figures where all curves are superimposed upon one another show a greater variation in the trials where either individuals with DNA added to the medium (orange) or groups of individuals (red) experienced desiccation events. The mean number ± standard deviation of eggs laid by the F1 generation as well as the number of individuals laying at least one egg (in brackets) are displayed in each histogram.

Importantly, the DNA taken up by *P*. *roseola* individuals appears to have consequences for the reproductive output of the untreated F1 generation, suggesting that it is also incorporated in the genome of the germ-line cells and is therefore heritable. Using the number of eggs laid as a proxy for fitness, there was significantly higher variation in the number laid by the F1 generation of *P*. *roseola* individuals desiccated with DNA than by those where the parents were desiccated without DNA (*F* = 1.853, *df =* 46,43, *P* = 0.043). Significantly higher variation in the former group also existed compared with the F1 generation of parents that were continuously hydrated with (*F* = 1.832, *df =* 46,76, *P* = 0.016) or without DNA (*F* = 2.166, *df =* 46,84, *P* = 0.003). No significant differences existed among the latter three treatments (hydrated vs. hydrated with DNA: *F* = 0.846, *df =* 76,84, *P* = 0.458; hydrated vs. desiccated: *F* = 0.855, *df =* 76,43, *P* = 0.545; and hydrated with DNA vs. desiccated: *F* = 1.012, *df =* 84,43, *P* = 0.988; Figure 3A). In addition, there was no effect of the desiccation procedure (generalized linear model (GLM), *T* = -0.870, *df =* 1,290, *P* = 0.385), the addition of DNA (*T* = 0.127, *df =* 1,290, *P* = 0.899) or their interaction (*T* = 0.779, *df =* 1,290, *P* = 0.437) on the mean number of eggs laid (Figure 1 and Figure 3).

### 3.3. Differential Effects between Desiccation in Groups Versus in Isolation

Moreover, the fitness effects were also present under more natural conditions. When the parental generation was desiccated in groups of individuals, the variance in the number of eggs laid by their untreated progeny was significantly higher than when the individuals were desiccated in isolation (*F* = 1.968, *df =* 66,66, *P* = 0.007) or were continuously hydrated (*F* = 1.915, *df =* 66,82, *P* = 0.005). Again, there was no difference between the latter two groups (*F* = 1.028, *df =* 66,82, *P* = 0.914; Figure 3B). By contrast, the mean of the reproductive output of the F1 generation of *P*. *roseola* desiccated individually was significantly lower than for that of the other treatments (GLM using Tukey contrasts: *F* = 20.22, *df =* 2,213, *P* < 0.0001): vs. hydrated *T* = −6.028, *P* < 0.0001; and vs. desiccated in groups *T* = −4.887, *P* < 0.0001. There was no difference between the mean number of eggs laid by the progeny of continuously hydrated parents and those desiccated in groups (*T* = −0.987, *P* = 0.664).

## 4. Discussion

Our results support the hypothesis of Boothby et al. [17] that animals able to undergo anhydrobiosis might also show an increased capacity to take up environmental DNA. In particular, the bdelloid rotifer *P*. *roseola* readily internalizes environmental genetic material with a strong preference toward DNA that is more similar to its own. This latter fact, together with the failure of the monogonont rotifer *B*. *rubens* to internalize any environmental DNA whatsoever, rules out the measured radioactivity stemming solely from the digestive tract of *P*. *roseola*, either from ^32^P from degraded DNA or ingested bacteria that themselves have ingested the labelled DNA because both processes would effectively “anonymize” the DNA such that no differential retention would be observed. However, it cannot be ruled out that bacteria might be the vector by which the DNA crosses the gut wall (see [37]). Moreover, the heritable effects we observed in the F1 generation indirectly support the incorporation of the environmental DNA in the genome in association with a desiccation event, leading to the on-going HGT events inferred for bdelloid rotifers [11,24].

Importantly, our results do not automatically imply the transfer of entire genes or only of genes of foreign (e.g., non-eukaryotic) origin. Previous studies (e.g., [11,25,26,32]) were necessarily biased in this direction because only genes from distantly related species can be detected easily using sequence-based analyses of the genome or transcriptome. The same mechanisms that facilitate both these ancient and on-going HGT events could also lead to the genetic exchange of gene fragments from more closely related species (as potentially implied in [30,31]). Indeed, the significantly longer retention times for conspecific environmental DNA we documented for *P*. *roseola* as well as the observed fitness effects when *P*. *roseola* were desiccated in groups rather than in isolation indicate that this process might be more common than otherwise indicated.

This process also appears to contribute to indirect (and infrequent?) genetic exchange in that the foreign DNA can also be transferred vertically to the offspring. Here, the fitness effects we observed agree with sexual theory, which predicts that the breakdown of existing genetic associations through the transfer of genetic material should alter the variance of traits in the following generation more strongly than their means [35,36]. For example, good alleles (or fragments thereof) are potentially released from bad genetic backgrounds (e.g., via deleterious mutations accumulated during the asexual phase) to be taken up in another, “better” genome. At the other extreme, bad alleles can find their way into bad genetic backgrounds. The resulting increase in variance in the subsequent generation thereby provides greater diversity for selection to act upon (visualized here as differential reproductive output in the untreated F1 generation).

Other potential explanations for our observed fitness effects can be ruled out through the experimental design. For instance, the result is unlikely to be a general effect of desiccation (e.g., potential errors during reconstruction of the genome; disruption of the hydrated, “active” life cycle; or epigenetic change; see [38,39,40]) because it would also have been present in the individuals of *P*. *roseola* desiccated in isolation that were used as controls. Similarly, any density-dependent effects from the higher density of the group treatment, albeit short in duration, would mean that the same fitness effects should not have been observed for animals desiccated individually with DNA added to the medium. At best, the added DNA could be viewed as a supplemental food source, thereby potentially increasing the mean of the reproductive output but not its variance. A similar increase in the mean would be expected if our results were a result of maternal effects, a process that has been implicated to be both widespread among and important for rotifers [41].

Instead, the general mechanism outlined by both Boothby et al. [17] and Eyres et al. [11] appears more likely. Desiccation disrupts the genome creating the double-stranded breaks [23] required to incorporate foreign genetic material during the rehydration process through a combination of the cell membranes being leaky to large macromolecules [18,19,20], the well-developed DNA repair mechanisms in bdelloids [23,32], and the scaffold provided by their degenerate tetraploid genome [42,43], all of which would facilitate the accidental incorporation of environmental DNA similar to the host genome. For the effects of the environmental DNA to be heritable, it needs to be incorporated in the genomes of the germ-line cells, but desiccation also disrupts the additional membrane that delimits these cells from the soma [44] and double-stranded breaks during desiccation are as common in oocytes as in somatic cells [22]. In this context, it is also worth noting that 37-nm nanoparticles can pass through the gut membranes and are in fact accumulated in the amictic eggs in the monogonont rotifer *Brachionus manjavacas* to be translated vertically to the offspring [45]. Because this species cannot withstand desiccation, the nanoparticles must pass through at least two intact cell membranes (possibly via either membrane pores or epithelial phagocytosis [45]), potentially documenting a general ability for ingested foreign particles to enter the somatic and germ-line cells in Rotifera. Implied in the above is that genomic incorporation of the foreign DNA requires that it already be present within the animals before the desiccation event so as to enter the germ-line cells. During the reanimation phase, the genome is already being reconstructed and, as our results show, less DNA is taken up during this time compared with constantly hydrated individuals, further reducing the chance of it becoming part of the genome.

This mechanism of genetic exchange, however, is relatively unspecific and appears to depend on the degree of similarity between the donor and host DNA. Such filtering is probably sufficient to avoid many of the negative effects associated with incorporating highly divergent DNA (e.g., interference with the needed repair of the double-stranded breaks inflicted by desiccation [21,42,43,46,47]) and would be strengthened by other, indirect mechanisms that promote exchange with more “advantageous” donors [46,48]. Foreign environmental DNA could still be incorporated depending on its similarity with the host DNA, which would explain the presence of ancient, alien DNA in the bdelloid genome as more of an accidental instance of HGT. By contrast, the greater sequence similarity of bdelloid DNA in general explains both why *P*. *roseola* incorporated DNA from *A*. *ricciae* as often as conspecific DNA as well as the observations that otherwise divergent bdelloid species often share several virtually identical alleles [30,31] and of apparent allele sharing in the bdelloid *Macrotrachela quadricornifera* [28].

Finally, the group desiccation trials indicate that, in addition to environmental DNA, bdelloid rotifers themselves could act as DNA sources during desiccation events. The resulting genetic exchange could thereby act to maintain low levels of gene flow within a species (including allele sharing; [28]) and thus its integrity as a species (see [9]). Although it is unclear if the donor DNA is released through the normal cell apoptosis associated with desiccation and/or with the death of individuals, it is noteworthy that genes associated with apoptosis, and transportation and translation, among others, are upregulated in *A*. *ricciae* 24 h after the onset of dehydration [25]. For our observed fitness effects, DNA would have to be released and taken up before desiccation was complete to be incorporated in the genome during its reconstruction in the reanimation phase. Supporting this hypothesis is the observation that reanimated individuals took up smaller amounts of environmental DNA, consistent with a reduced DNA uptake during this time when the priority is to reconstitute both the genotype and phenotype. Ecologically, this timing also ensures abundant amounts of conspecific DNA in the limited amount of water present near the end of the desiccation process (e.g., individuals of *P*. *roseola* tend to stay together if desiccated and also while laying eggs), something that is more difficult to achieve under normal, favorable conditions.

Our observations of the active uptake of DNA fragments by *P*. *roseola*, their (passive?) filtering according to the relatedness of the donor species, as well as their heritable effects point to a potential mechanism for the regular, if limited (contra [30,31]), genetic exchange in bdelloid rotifers that has only essentially been observed *a posteriori* by other authors until now (e.g., [11,24,25,26,32]). It remains to be investigated how universal these results are across bdelloids and how they apply in particular to those few species that do not undergo anhydrobiosis (cf. [11]). If this or similar mechanisms do indeed exist across the group, bdelloids would serve as yet another, but extremely high-profile, case study where the “cryptic” exchange of genetic material was found in a supposedly purely asexual lineage (e.g., the parasitoid wasp *Lysiphlebus* [49]; the aphid *Tramini* [50]; the fungi *Candida*, *Aspergillus* [51] and Glomeromycota [52]; the amoeba *Entamoeba histolytica* [53]; and colpodean ciliates [54]) (see also [55]). Even the HGT found throughout the otherwise asexual bacteria has been held to be an important mechanism driving adaptive diversity in these organisms [27], if not more widely across metazoans than is commonly assumed [55]. Altogether, these findings in combination with our results and those of others documenting on-going HGT in bdelloid rotifers [11,24] reinforce the notion that some form of genetic exchange appears to be necessary for the long-term survival of otherwise largely asexual species.

## Figures and Tables

**Table 1:** Pairwise comparisons of radioactive, environmental DNA derived from different rotifer species taken up by *Philodina roseola* compared with the reference of *P. roseola* DNA taken up by hydrated *P. roseola*. Statistical tests compared both the number of trials in Which DNA Was Taken up as well as the amount taken up in terms of amount of radioactivity measured (see also Figure 2).

*Philodina roseola* Treatment	Environmental DNA Source Species	Incubation Time [Hours]	Paired *t*-Test (Two-Sided) of the Amount of Radioactive DNA Taken up	Contingency Table for Proportion of Trials in Which DNA Was Taken up (*df* = 1 in All Cases)
Desiccated	*P. roseola*	3	*t* = 2.416, *df* = 9, *P* = 0.039	7/10, χ^2^ = 3.529, *P* = 0.060
		15	*t* = 4.352, *df* = 29, *P* < 0.001	30/30, χ^2^ = 0, *P* = 1.000
Hydrated	*B. rubens*	1.5	*t* = −0.725, *df* = 14, *P* = 0.480	14/15, χ^2^ = 1.035, *P* = 0.309
		15	*t* = 3.855, *df* = 14, *P* = 0.002	7/15, χ^2^ = 10.909, *P* < 0.001
	*E. dilatata*	15	*t* = 2.333, *df* = 14, *P* = 0.035	8/15, χ^2^ = 9.130, *P* = 0.003
	*A. ricciae*	15	*t* = 0.589, *df* = 14, *P* = 0.565	13/15, χ^2^ = 2.143, *P* = 0.143
